# Analyses of Food

**Published:** 1883-03

**Authors:** 


### ANALYSES OF FOOD.

In a long and interesting article in the *Pharmaceutische Centralhalle* on the nourishing powers of various natural and artificial foods for infants and invalids, Dr. Stutzer, of Bonn, gives the following results as far as concerns their nitrogenous constituents :

| | Flesh Formers, per cent. | | Flesh Formers, per cent. |
|---|---|---|---|
| Caviare | 25.81 | Condensed milk | 8.79 |
| Ervalenta | 19.93 | White bread | 7.20 |
| Smoked ham | 18.92 | Biscuit | 6.71 |
| Fresh beef | 18.53 | Oysters | 5.78 |
| Fowl (breast) | 16.56 | Cow's milk | 4.00 |
| White of egg | 13.48 | Extractum carnis | 3.40 |
| Yolk | 13.01 | Malt extract | 0.28 |
| Infants' food | 9.90 | | |

The above table gives rise to some curious reflections. The wonderful nourishing powers attributed to oysters are found to dwindle into insignificance when compared with other food; for instance, a single hen's egg contains as much nourishment, that is to say, as much flesh-forming material, as fourteen oysters, while a quarter of a pound of lean rump steak is equal to about five dozen of these delicious but delusive mollusks.

With regard to condensed milk, it contains much less flesh-forming material than is generally supposed. Taking four per cent. for cow's milk as a fair average, the directions on the cans, if followed out, give unexpected results. For children's use, we are told to dilute the condensed milk with four or five parts of water. Taking the lowest figure, we should then have five parts of diluted condensed milk, which, according to Dr. Stutzer's figures, would only contain 1.76 per cent. of flesh formers, instead of four per cent., while the milk sugar would be increased from 4.5 to 10.85 per cent. We know that woman's milk contains more sugar than cow's, but still not in the above surprising proportions. Now that so much canned milk is used for infants brought up by hand, it becomes a question how far mothers who cannot suckle their children are responsible for the health, and even lives, of their children, by giving them milk from the tin cow instead of that of the living animal.

Dr. Stutzer's figures also further expose the often-exposed superstition about the nourishing powers of beef tea. He extracted all the extractible matter from 100 grammes of beef with 100 grammes of water, and a good proportion of salt, at a gentle heat for four hours, but could only succeed in obtaining in solution one twelfth of the nourishing matter of the beef, the remaining eleven-twelfths remaining behind in the *bouilli.* In other words, we should have to take half a gallon of beef tea made with a pound of beef and a pint of water before we got as much nourishment as is contained in a quarter of a pound of steak. We might, it is true, evaporate our beef tea down to, say, half a pint, but we doubt if it would be palatable to the least squeamish invalid. The high value of eggs, too, is well shown; in fact, roughly speaking, a couple of eggs weighing three and a half ounces are about equal to two ounces of good rump steak.

Dr. Stutzer, in the course of his article, mentions three samples of cocoa warranted free from fat (*ent[illegible]lter Cacao*), from different houses, which contained, respectively, 33.48, 32.31, and 30.95 per cent. of fatty matter of some sort !

The highly nourishing powers of caviare will no doubt strike the "general" with amazement.

---

THE MUNGOOSE AS A RAT KILLER.—The introduction of the mungoose into Jamaica as a cure for the once formidable rat pest on the sugar plantations is said to have proved a notable success. The sugar rat is a huge, white-bellied fellow, measuring ten inches in length of body, his long tail adding ten inches more to his length. Formerly the damage done to the sugar plantations of the island by these rats amounted to something like half a million dollars a year, rising to a quarter of the crop in seasons of special ravages. About five years ago the mungoose, whose zeal as a snake and rat killer is well known, was imported from India. As a result, the plague of rats has been greatly diminished, with a saving in sugar of not less than 25 tons of sugar on each estate. There is also saved the expense of rattage, formerly amounting to hundreds of dollars a year

